# Maximal respiratory static pressures in patients with different stages of COPD severity

**DOI:** 10.1186/1465-9921-9-8

**Published:** 2008-01-21

**Authors:** Claudio Terzano, Daniela Ceccarelli, Vittoria Conti, Elda Graziani, Alberto Ricci, Angelo Petroianni

**Affiliations:** 1 Department of Cardiovascular and Respiratory Sciences, UOC Malattie Respiratorie, University of Rome "La Sapienza", Italy

## Abstract

**Background:**

In this study, we analyzed maximal inspiratory pressure (MIP) and maximal expiratory pressure (MEP) values in a stable COPD population compared with normal subjects. We evaluated the possible correlation between functional maximal respiratory static pressures and functional and anthropometric parameters at different stages of COPD. Furthermore, we considered the possible correlation between airway obstruction and MIP and MEP values.

**Subject and methods:**

110 patients with stable COPD and 21 age-matched healthy subjects were enrolled in this study. Patients were subdivided according to GOLD guidelines: 31 mild, 39 moderate and 28 severe.

**Results:**

Both MIP and MEP were lower in patients with severe airway impairment than in normal subjects. Moreover, we found a correlation between respiratory muscle function and some functional and anthropometric parameters: FEV_1 _(forced expiratory volume in one second), FVC (forced vital capacity), PEF (peak expiratory flow), TLC (total lung capacity) and height. MIP and MEP values were lower in patients with severe impairment than in patients with a slight reduction of FEV_1_.

**Conclusion:**

The measurement of MIP and MEP indicates the state of respiratory muscles, thus providing clinicians with a further and helpful tool in monitoring the evolution of COPD.

## Background

In several diseases, the evaluation of respiratory muscle strength can prove to be very useful. The measurement of the maximum static mouth pressures made against an occluded airway (maximal expiratory pressure and maximal inspiratory pressure) is the most widely used and is a simple way to gauge respiratory muscle strength and to quantify its severity [1-3].

When we analyze maximal respiratory pressure, we should consider both the difficulty that some subjects have in performing a maximal effort and the normal biological variability of respiratory muscle strength [4].

Maximal inspiratory pressure (MIP) is the maximum negative pressure that can be generated from one inspiratory effort starting from functional residual capacity (FRC) or residual volume (RV) [5,6]. Maximal expiratory pressure (MEP) measures the maximum positive pressure that can be generated from one expiratory effort starting from total lung capacity (TLC) or FRC. Unlike inspiratory muscles, expiratory muscles (abdominal and thoracic muscles) reach their optimal force-length relationship at elevate pulmonary volumes [7].

During normal breathing, most of the respiratory work depends on the diaphragm function and the accessory respiratory muscles become necessary only during deep inspiration [8].

The mouth pressures recorded during these maneuvers are assumed to reflect respiratory muscle strength [9].

It is known that a reduction of MIP and MEP has been associated with several neuromuscular diseases, but it is also possible to point up lower values in patients with chronic obstructive pulmonary disease (COPD) [10-12].

The factors contributing to respiratory muscle weakness in many patients with COPD are: a) malnutrition related to biochemical, anatomical and physiological changes; b) muscular atrophy; c) steroid-induced myopathy; d) pulmonary hyperinflation with increased residual volume; e) reduced blood flow to the respiratory muscles [13-19].

The measurement of MIP and MEP is indicated in any of these situations or when dyspnea or hypercapnia are not proportional to FEV_1 _reduction [6].

Age and sex could influence MIP and MEP values; these are lower in females than in males and quite constant until seventy years of age when they start to decrease [6].

The objectives of this study were: (1) to describe MIP and MEP values in a stable COPD population, (2) to explore the effect of varying degrees of obstructive ventilatory impairment on MIP and MEP measurement, (3) to evaluate the possible correlation between functional maximal respiratory pressures and functional parameters at different stages of COPD.

## Methods

During 1 year 110 patients suffering from COPD were enrolled in this study. All patients were in a clinically stable condition and the mean age was 70 ± 8 years (Table 1).

**Table 1:** Characteristics of patients

	**CONTROL GROUP**	**MILD**	**MODERATE**	**SEVERE**	p VALUE*	**COPD PATIENTS**
**N°**	21	31	39	28		98
**Age**	67 ± 5	67 ± 8	71 ± 6	72 ± 7	p > 0.05	70 ± 8
**Body weight (Kg)**	72 ± 6	76 ± 11	78 ± 12	75 ± 20	p > 0.05	77 ± 15
**Body height (cm)**	165 ± 8	168 ± 9	166 ± 8	167 ± 6	p > 0.05	167 ± 8
**MIP (cmH**_2_**O)**	99 ± 18	84 ± 22	80 ± 34	65 ± 20	p = 0.0002	77 ± 28
**MEP (cmH**_2_**O)**	102 ± 26	93 ± 29	90 ± 32	75 ± 21	p = 0.01	89 ± 31
**FEV_1 _(L)**	2.7 ± 0,5	2.4 ± 0,6	1.5 ± 0.4	0.9 ± 6.3	p < 0.0001	1.7 ± 0.7
**FEV_1 _%**	102 ± 12	91 ± 11	65 ± 8	38 ± 7	p < 0.0001	65 ± 22
**FVC (L)**	3.3 ± 0.9	3.2 ± 0.8	2.4 ± 0.7	1.9 ± 0.6	p < 0.0001	2.5 ± 0.9
**FVC %**	101 ± 12	96 ± 9	76 ± 10	59 ± 14	p < 0.001	77 ± 18
**PEF (L/sec)**	6.5 ± 2	6.6 ± 1.6	4.9 ± 1.7	3.1 ± 1.2	p < 0.0001	4.9 ± 2
**PEF %**	102 ± 12	91 ± 15	71 ± 18	45 ± 15	p < 0.0001	70 ± 24
**TLC (L)**	4.9 ± 1.3	5.9 ± 1.1	5.4 ± 1.3	5.6 ± 1.4	p = 0.037	5.7 ± 1.3
**TLC %**	98 ± 20	98 ± 13	94 ± 14	97 ± 18	p = 0.0007	97 ± 16
**RV (L)**	2 ± 0.5	2.5 ± 0.7	3 ± 0.8	3.9 ± 1.2	p < 0.0001	3 ± 1
**RV %**	97 ± 20	111 ± 29	124 ± 32	151 ± 47	p < 0.0001	128 ± 39
**RV/TLC %**	40 ± 20	41 ± 11	50 ± 20	66 ± 23	p < 0.0001	49.7 ± 19

*Comparison between the four groups (control, mild, moderate, severe) have been made by analysis of variance (ANOVA).

At the first examination, we excluded those patients whose FEV_1 _improvement, after a bronchodilatory test, was ≥ 12% and 200 mL of the baseline value and with a history of asthma. Other patients, with clinically significant diseases such as fibrothorax, bronchiectasis, tuberculosis or neuromuscular diseases were also excluded. Some patients were excluded because of lack of compliance during the forced expiratory test or during the MIP and MEP maneuvers.

All patients gave their written informed consent before the study and the Ethics Committee approved the protocol.

At the enrolment, all the subjects were examined. Anthropometric measurements (age, height and weight) were taken and pulmonary function tests, including flow/volume spirometry and N2 Washout, were conducted. Maximal static inspiratory and expiratory mouth pressures were measured using a portable mouth pressure meter (Spirovis – COSMED – Pavona – Italy); this had a disposable mouthpiece, and a small leak to prevent glottic closure. MIP was obtained at the level of RV and MEP was measured at the level of TLC. The measurements were made in standing position. The subjects were verbally encouraged to achieve maximal strength. The measurements were repeated until five values varying by less than 5% and sustained for at least 1 s were obtained; the best value achieved was considered in the data analysis.

Forced expiratory tests were recorded with a Cosmed Quark spirometer (PFT4 SUITE – COSMED – Pavona – Italy) which was calibrated each morning using the same 3 L precision syringe.

We divided patients into three groups on the basis of airway obstruction: mild (FEV1 > 80%), moderate (FEV1 between 80 and 50%), and severe (FEV1 < 50%) with FEV1/FVC ratio <70% in all the groups.

The control group included 21 age-matched normal subjects who were non-smokers, free of respiratory symptoms and disease and with normal functional parameters (Table 1).

Statistical analysis was performed for all measured functional parameters using GraphPad Prism version 4.00 for Windows (GraphPad Software, San Diego California USA). Standards descriptive statistics based on mean and standard deviation (SD) for quantitative variables were used. The mean difference between MIP/MEP in healthy subjects and MIP/MEP in patients with COPD was determined by the unpaired Student's t test. Statistical differences for respiratory muscle strength and different stage of COPD were analyzed by analysis of variance (ANOVA). The Pearson's test was performed to assess possible correlation between MIP and MEP values and anthropometric and functional parameters.

All p values were two sided and values below 0.05 were considered statistically significant.

## Results

During this study, 12 COPD patients were withdrawn: 5 because of a contemporary restrictive deficit and 7 because of their insufficient compliance during the forced expiratory test or during the MIP and MEP maneuvers. Therefore, 98 patients took part in this study. Table 1 shows the anthropometric parameters and lung function of the patients studied.

The MIP and MEP values of healthy subjects were used as a control group for the comparison with patients with different stages of COPD.

MEP was significantly lower (p = 0.0014) in patients with severe airway obstruction than in the control group; no differences were observed in mild and moderate patients (p > 0.05).

At the same time, MIP was significantly lower at all the stages of COPD than in the control group: mild p = 0.010; moderate p = 0.018; severe p < 0.0001.

**Figure 1:**
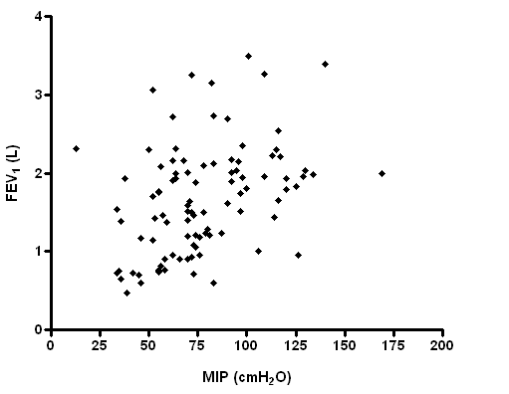
Relationship between MIP and FEV_1 _(p = 0.0002; r^2 ^= 0.13).

Statistical analysis performed on the total of COPD patients to assess the possible correlation between MIP and MEP values and anthropometric and functional parameters showed significant (p < 0.05) positive correlations among maximal static inspiratory pressure and FEV_1 _(L, r^2 ^= 0.13, Figure 1), FVC (L, r^2 ^= 0.20), PEF (L/sec, r^2 ^= 0.19), TLC (L, r^2 ^= 0.11) and height (r^2 ^= 0.09).

**Figure 2:**
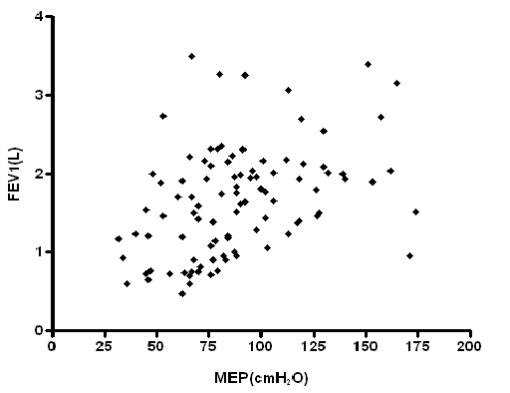
Relationship between MEP and FEV_1 _(p = 0.0002; r^2 ^= 0.13).

Similar results were showed between MEP and the same functional and anthropometric parameters (FEV1 r^2 ^= 0.13, figure 2; FVC r^2 ^= 0.19; PEF r^2 ^= 0.22; TLC r^2 ^= 0.12; height r^2 ^= 0.15).

**Figure 3:**
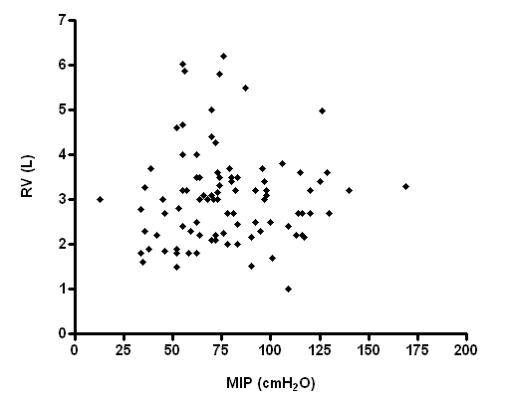
Relationship between MIP and RV (p > 0.05; r^2 ^= 0.01).

**Figure 4:**
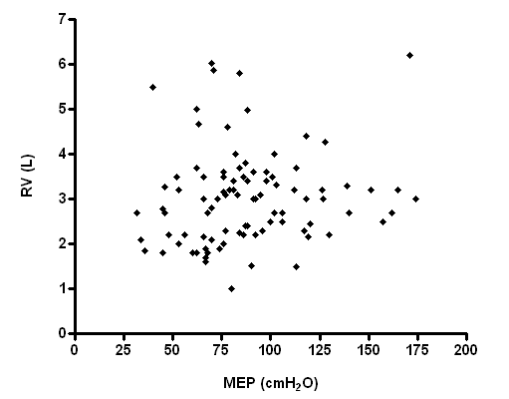
Relationship between MEP and RV (p > 0.05; r^2 ^= 0.0009).

**Figure 5:**
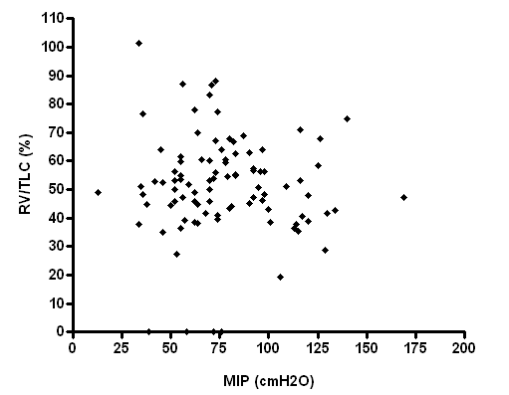
Relationship between MIP and RV/TLC (p > 0.05; r^2 ^= 0.01).

**Figure 6:**
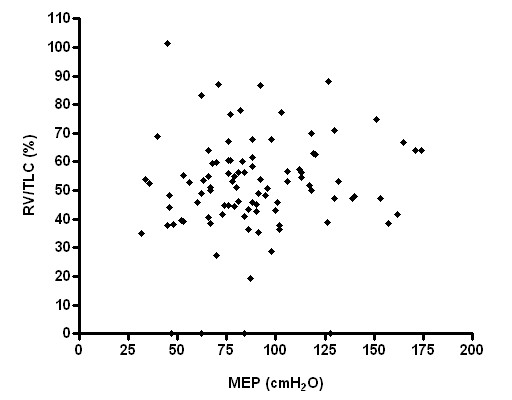
Relationship between MEP and RV/TLC (p > 0.05; r^2 ^= 0.01).

Respiratory muscle strength, however, had no significant correlation with RV and RV/TLC (Figures 3, 4, 5, 6), weight and age.

Moreover, we evaluated whether there was a possible correlation between COPD stage and respiratory muscular strength. The analysis of variance (ANOVA) showed no statistically significant difference between mild and moderate patients (p > 0.005), while the difference between mild and severe was significant as well as that between moderate and severe patients.

## Discussion

To our knowledge, this is the first study that analyzes MIP and MEP variation in the different stages of COPD severity to understand when MIP and MEP start to decrease.

The main finding of this work is that airway obstruction may be closely associated with decreased respiratory pressures in patients with COPD. In fact, both MIP and MEP values were lower in patients with severe obstruction compared with healthy subjects. MIP decreased also in patient with mild and moderate functional impairment: this could suggest an earlier deterioration of the inspiratory muscles in this sort of patients.

Similar results have been showed in a recent work that limited the evaluations only to the inspiratory muscle strength [20].

Dynamic functional parameters are a measure of the respiratory muscular strength as well as maximal respiratory pressures. In fact with the reduction of FEV_1_, PEF and FVC, as in severe COPD, we have observed a similar decrease in MIP and MEP. Interestingly, our study highlights the importance of the predictivity of functional parameters (FEV_1_, PEF, FVC, and TLC) on MIP and MEP reduction in COPD patients.

Nishimura and colleagues showed a similar relation between respiratory muscle force and FEV_1 _[18].

Our findings could be explained by several factors. Patients with severe disease probably have a decrease in tension produced by inspiratory muscle shortening. However, our study did not show a correlation between maximal respiratory pressure and residual volume at any of the different stages even though air-trapping was different among patients.

As reported by Rochester, chronic airflow limitation permanently shortens the diaphragm, in this way muscles lose sarcomeres, but the length of individual sarcomeres is restored to normal and the force-length relationship of the shortened diaphragm is reset to a new and shorter muscle length so, the relation of diaphragm length to lung volume is the same as in normal subjects [12].

Further support for the concept that COPD does not permanently alter diaphragm muscle length in human COPD comes from another study by the same author who reported that the diaphragm undergoes an adaptation to compensate for the mechanical stresses that pulmonary hyperinflation places on it [21].

Similarly, Nishimura and colleagues showed no significant correlation between respiratory muscular strength and RV [18].

Similowsky et al. demonstrated that the diaphragm of COPD patients undergoes some structural adaptation which preserves or even increases its capacity to generate pressure even if the muscular function is impaired because of an alteration in chest wall geometry [22,23].

Walsh and coworkers found that the size of the rib cage and the arrangement of the ribs where not different between severely hyperinflated patients with COPD and healthy subjects [24].

McKenzie et al observed that at resting functional residual capacity the curvature of the diaphragm is only 3.5% smaller in patients with severe COPD than in healthy subjects [25].

Additional mechanisms of muscular impairment in COPD may include malnutrition, which predisposes the diaphragm to a greater loss in muscle mass in proportion to a patient's body-weight reduction [26-30]. Prolonged malnutrition can lead to skeletal and respiratory muscle wasting with severe effects on the contractile and fatigue properties of the diaphragm [26-30]. This suggests that a nutritional supplementation should be a primary intervention in patients with lean body mass [31].

Corticosteroids, routinely used to manage chronic inflammation, have negative consequences, including steroid myopathy of respiratory and skeletal muscles, even at low doses [26-28].

Further, electrolyte imbalance and hypoxemia alter muscle function and should be corrected, when possible [26,27].

Oxidative stress, disuse, and systemic inflammation may contribute to the observed muscle abnormalities and each factor has its own potential for innovative treatment approaches [26,27].

These processes contribute to the reduced capacity of the respiratory muscles in COPD and translate to measurable decreases in maximal pressure generation, exhibited as lower values for maximal inspiratory pressure (MIP), maximal expiratory pressure (MEP), sniff testing, maximal voluntary ventilation (MVV), and exercise tolerance.

There is also a strength correlation between thoracic morphology dimension and anthropometric variables even if some studies have obtained conflicting results with age, weight and height [32].

Wilson and co-workers reported that MIP and MEP in men were significantly correlated with age and weight, whereas in women they were correlated with height and weight, while Leech et al found that age had no consistent effect on respiratory muscular strength [33,34].

In contrast with the study of Enright et al [35], our study did not find any correlation with weight and age, probably because our patients had reached approximately 60 years of age with the same normal body weight.

For these reasons our study shows a significant linear relationship between respiratory muscles pressure and height. This is likely to reflect an association between stature, diaphragm position and inspiratory muscle strength.

Nishimura showed that only lean body mass and abnormal body weight were associated with decreased respiratory strength [18].

To conclude, when we treat a patient with severe airflow obstruction we should consider the possible respiratory muscle deterioration that could affect this sort of patients. The periodical evaluation of the respiratory muscle strength could represent a further and helpful tool in monitoring the disease severity.

Our study is only a "snapshot" of the maximal respiratory pressures and their correlation with functional parameters at different stages of COPD severity in a hundred patients living in Rome.

Further studies on a larger population sample are needed to confirm our result.

## Abbreviations

**COPD: **Chronic Obstructive Pulmonary Disease

**FEV_1_: **Forced Expiratory Volume in one second

**FRC: **Functional Residual Capacity

**FVC: **Forced Vital Capacity

**MIP: **Maximal Inspiratory Pressure

**MEP: **Maximal Expiratory Pressure

**PEF: **Peak Expiratory Flow

**RV: **Residual Volume

**TLC: **Total Lung Capacity

**VC: **Vital Capacity

## Competing interests

The author(s) declare that they have no competing interests.

## Authors' contributions

CT conceived the trial, participated in its design, study procedures, interpretation of results, performed the statistical analysis and helped to draft the manuscript. DC participated in the study procedures, in its design, interpretation of results, performed the statistical analysis and helped to draft the manuscript. VC participated in the study procedures, interpretation of results and helped to draft the manuscript. EG participated in study design and helped to draft the manuscript. AR participated in the study procedures and helped to draft the manuscript. AP participated in study design, study procedures, interpretation of results and helped to draft the manuscript. All of the authors read and approved the final manuscript.
